# Design and Performance Evaluation of a Dual Antenna Joint Carrier Tracking Loop

**DOI:** 10.3390/s151025399

**Published:** 2015-10-01

**Authors:** Wenfei Guo, Tao Lin, Xiaoji Niu, Chuang Shi, Hongping Zhang

**Affiliations:** 1 GNSS Research Center, Wuhan University, Wuhan 430079, China; E-Mails: wf.guo@whu.edu.cn (W.G.); shi@whu.edu.cn (C.S.); hpzhang@whu.edu.cn (H.Z.); 2 Department of Geomatrics Engineering, University of Calgary, Calgary, AB T2N 1N4, Canada; E-Mail: tlin@ucalgary.ca

**Keywords:** GNSS receiver, carrier phase tracking, differential carrier phase, weak signal, loop bandwidth

## Abstract

In order to track the carrier phases of Global Navigation Satellite Systems (GNSS) signals in signal degraded environments, a dual antenna joint carrier tracking loop is proposed and evaluated. This proposed tracking loop processes inputs from two antennas, namely the master antenna and the slave antenna. The master antenna captures signals in open-sky environments, while the slave antenna capture signals in degraded environments. In this architecture, a Phase Lock Loop (PLL) is adopted as a master loop to track the carrier phase of the open-sky signals. The Doppler frequency estimated by this master loop is utilized to assist weak carrier tracking in the slave loop. As both antennas experience similar signal dynamics due to satellite motion and clock frequency variations, a much narrower loop bandwidth and possibly a longer coherent integration can be adopted to track the weak signals in slave channels, by utilizing the Doppler aid from master channels. PLL tracking performance is affected by the satellite/user dynamics, clock instability, and thermal noise. In this paper, their impacts on the proposed phase tracking loop are analyzed and verified by both simulation and field data. Theoretical analysis and experimental results show that the proposed loop structure can track degraded signals (*i.e.*, 18 dB-Hz) with a very narrow loop bandwidth (*i.e.*, 0.5 Hz) and a TCXO clock.

## 1. Introduction

Carrier phase tracking is an important but very vulnerable operation in a GNSS receiver, due to the short GNSS carrier wavelength and the low transmitted signal power [1,2]. Utilizing a narrow loop bandwidth is one solution for tracking weak carrier phases. However, the bandwidth should be wide enough to accommodate the satellite/receiver dynamics and the clock frequency variations to avoid loss of locks [3]. Thus, in a standalone GNSS receiver, the loop bandwidth value is a compromise choice between sensitivity and signal dynamics [4].

In order to reduce the loop bandwidth for weak signal tracking, various Doppler aiding methods have been proposed [5]. One of the most common methods is vector tracking [6,7], which tracks signals of all satellites jointly and allows aiding among satellites for more robust signal tracking. Another technology is GNSS/Inertial Navigation System (INS) deep integration. The key of this technology is to reduce the carrier phase tracking loop bandwidth by utilizing the received/satellite contributed Doppler estimates from a GNSS/INS integrated navigation solution [8,9]. This integration method is very suitable for highly dynamic or strong interference applications [10]. However, inertial measurement units (IMUs) cannot provide good estimates on the receiver clock frequency instability [11], which is the key limiting factor of carrier tracking in low dynamic applications. To solve the problem, a dual antenna GNSS tracking loop was designed. This tracking loop has two inputs. One is from the master antenna in open sky, while the other one is from the slave antenna on the point to be measured. Master tracking loops are used for strong signal tracking and provide Doppler assistance to slave tracking loops. Since master and slaves loops are driven by the same oscillator in the receiver, and master and slave loops are tracking the signals from the same satellites, the slave tracking loop can track signals with much reduced bandwidth for improved tracking sensitivity. In this paper, the theoretical model and performance analysis of this proposed carrier tracking loop are presented. The impacts of signal dynamics, clock phase noise and thermal noise on carrier phase tracking are analyzed. The applications of the proposed tracking loop for RTK are also introduced.

The paper is organized as follows: Section 2 recalls the carrier tracking principles and summarizes the impacts of signal dynamics, clock phase noise and thermal noise in carrier phase tracking. Section 3 presents the tracking loop structure of the dual antenna receiver, and analyzes the tracking errors of the slave tracking loop and differential carrier phase induced by different error sources. Section 4 gives the simulation results compared with the standard independent tracking architecture. Section 5 describes the test results of a software receiver using the new loop structure. Finally, some conclusions are drawn in the last section.

## 2. Carrier Tracking Loop and the Phase Error

**Figure 1:**
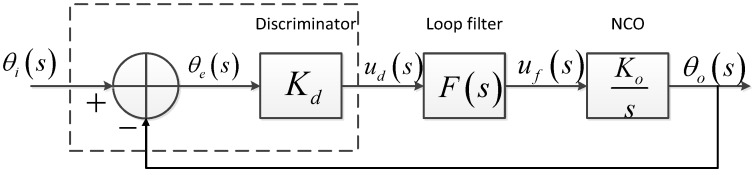
PLL Tracking Loop Structure.

As the signal acquisition process in a GNSS receiver only provides rough estimates of the Doppler frequency and code delay of the satellite signals in view [11], a circuit with a feedback loop named tracking loop is introduced following the acquisition in order to get more precise carrier and code phase estimates. A basic analog domain phase-locked loop structure is shown in Figure 1, including a phase discriminator, a loop filter and a local numerical controlled oscillator (NCO). The carrier tracking loop uses the In-phase and Quadrature channel integrator outputs to calculate the phase error, then a loop filter is introduced to eliminate the high frequency noise, and the result is used as a controlled quantity to tune the frequency of the carrier NCO.

The transfer function of the PLL above can be given as follows:

(1) $$H\left(s\right)=\frac{\theta_{o}\left(s\right)}{\theta_{i}\left(s\right)}=\frac{KF\left(s\right)}{s+KF\left(s\right)}$$

where $\theta_{i}\left(s\right)$ is the input signal phase, and $\theta_{o}\left(s\right)$ is the loop output phase, $K=K_{d}K_{o}$ represents the loop gain, *F*(*s*) is the loop filter, which decides the order of the loop. The transfer function of a frequently-used 3rd PLL loop is as follows:

(2) $$H\left(s\right)=\frac{2\omega_{n}s^{2}+2\omega_{n}^{2}s+\omega_{n}^{2}}{s^{3}+2\omega_{n}s^{2}+2\omega_{n}^{2}s+\omega_{n}^{3}}$$

Here $\omega_{\text{n}}$ is the natural frequency. The PLL output phase error mainly includes thermal noise $\sigma_{w}$, dynamic stress error e(t) (induced by signal dynamics), vibration-induced oscillator phase noise $\sigma_{v}$ and Allan deviation oscillator phase noise $\sigma_{A}$. Considering all these error sources, one metric that is normally used to determine if a PLL keeps in lock is whether the total phase jitter $\sigma_{\theta }$ satisfies the following expression:

(3) $$\sigma_{\theta \;}=\sqrt{\sigma_{w}^{2}+\sigma_{v}^{2}+\sigma_{A}^{2}}+\frac{e\left(t\right)}{3}<15{}^{\circ}$$

Each of the phase jitter components and their impact on the carrier tracking loop parameters is discussed in the following subsections.

### 2.1. Thermal Noise

Thermal noise exists in every system. It is a random signal with a constant power spectral density. Its effect on a system mainly depends on the PSD and the system bandwidth. However, most of the actual systems aren’t ideal, which means its amplitude response isn’t an ideal rectangle. In this case, the noise equivalent bandwidth is usually used to analyze the noise effect, which is defined by [12]:

(4) $$B_{L}=\frac{1}{2\pi {\left|H_{ideal}\left(\omega \right)\right|}_{G}^{2}}\overset{\infty }{\underset{0}{\int }}{\left|H\left(\omega \right)\right|}^{2}d\omega$$

where H(ω)  is the transfer function and ${\left|H_{ideal}\left(\omega \right)\right|}_{G}\;$ is the system gain of the ideal filter. Assuming the PSD of the thermal noise is *N*_0_/2, the output power of such a system can be obtained:

(5) $$P=\frac{N_{0}}{2}\cdot 2B_{L}{\left|H_{ideal}\left(\omega \right)\right|}_{G}^{2}=\frac{N_{0}}{2\pi }\overset{\infty }{\underset{0}{\int }}{\left|H\left(\omega \right)\right|}^{2}d\omega$$

The thermal noise phase effect for a PLL is determined by the carrier noise ratio and the loop bandwidth. In a GNSS receiver, the noise enters the PLL through the discriminator after pre-detection integration. The most frequently used atan(·) discriminator has the error variance as follows [13]:

(6) $$\theta_{e}^{2}=\frac{1}{2T_{coh}C/N_{0}}\left(1+\frac{1}{2T_{coh}C/N_{0}}\right)$$

where *T_coh_* is the coherent integration time and *C/N*_0_ is the carrier noise ratio. According to the definition of the equivalent noise bandwidth, the coherent integration could be seen as a low pass filter, whose two-sided bandwidth is 1/*T_coh_*, and system gain is 1. Thus, the noise Power Spectral Density can be obtained according the equation above:

(7) $$\frac{N_{0}}{2}=\frac{\theta_{e}^{2}}{1/T_{coh}}=\frac{1}{2C/N_{0}}\left(1+\frac{1}{2T_{coh}C/N_{0}}\right)$$

For a PLL noise equivalent bandwidth *B_L_*, the thermal noise phase jitter can be derived as follows under the assumption that the system gain is 1:

(8) $$\sigma_{t}^{2}=\frac{N_{0}}{2}\cdot 2B_{L}\cdot {\left|H_{ideal}\left(\omega \right)\right|}_{G}^{2}=\frac{B_{L}}{C/N_{0}}\left(1+\frac{1}{2T_{coh}C/N_{0}}\right)$$

From the equation above, it can be seen that the thermal noise phase jitter is a function of the coherent integration time *T_coh_* and the one-sided PLL loop bandwidth *B_L_*. Thus, in order to reduce the thermal noise phase jitter, the main methods are reducing the loop bandwidth and increasing the coherent integration time. However, the coherent time is limited by navigation bit, whose rate is 50 Hz for GPS, and the *B_L_* is limited by the signal dynamics due to satellite/receiver motion and clock frequency variations.

### 2.2. Dynamic Stress

The steady state phase tracking error which is induced by dynamic is known as the dynamic stress of a PLL. It can be represented as follows [2]:

(9) $$e\left(t\right)=\frac{2\pi }{\lambda }\frac{1}{\omega_{n}^{N}}\frac{d^{N}R}{dt^{N}}\left[\text{rad}\right]$$

where *R* is the distance between the satellite and the receiver, *N* is the loop order, and λ means wavelength of the carrier. It can be seen from the equation that 1st order loop is sensitive to velocity stress, 2nd order loop is sensitive to the acceleration stress, and 3rd order loop is sensitive to the jerk stress. The dynamic stress induced phase error increases as the characteristic frequenc $,\;\omega_{n}$ decreases. Thus, smaller bandwidth loop can lead to a lager dynamic stress phase error. This means that signal dynamic restricts the reduction of the loop bandwidth.

### 2.3. Clock Phase Noise

The vibration-induced oscillator phase noise $\sigma_{v}$ and the Allan deviation oscillator phase noise $\sigma_{A}$ are the two main types of clock noise phase jitter, which originates from the satellite and receiver clock phase noise. The contribution of the clock noise (correlated errors) to GNSS phase tracking loops can be determined by [14]:

(10) $$\begin{array}{l} \sigma_{c}^{2}=\frac{1}{2\pi }\overset{\infty }{\underset{0}{\int }}{\left|H_{e}\left(\omega \right)\right|}^{2}G_{c}\left(\omega \right)d\omega \\ =\frac{1}{2\pi }\;\overset{\infty }{\underset{0}{\int }}{\left|1-H\left(\omega \right)\right|}^{2}G_{c}\left(\omega \right)d\omega \\ =\frac{1}{2\pi }\overset{\infty }{\underset{0}{\int }}\frac{\omega^{2n}}{\omega_{n}^{2n}+\omega^{2n}}G_{c}\left(\omega \right)d\omega \end{array}$$

where $H_{e}\left(\omega \right)=1-H\left(\omega \right)$ is the error transfer function of the loop, and $G_{c}\left(\omega \right)$ is the Power Spectral Density (PSD) of the colored noises. Due to the high pass characteristic of the error transfer function of the loop in the equation, the clock noise phase jitter becomes larger with decrease of the loop bandwidth. Consequently, the impact of this error should be considered when reducing the loop bandwidth.

## 3. Structure and Phase Error Analysis of Dual Antenna Tracking Loop

**Figure 2:**
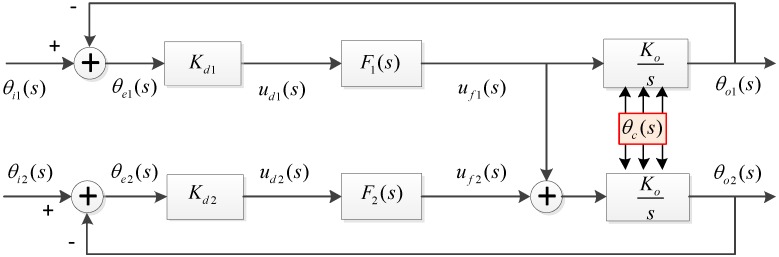
Tracking loop structure of the dual antenna receiver.

The proposed dual antenna tracking architecture utilizes the tracking information from master channels to help the signal processing in slave channels. Similar to A-GNSS, the information obtained from the master antenna could be used to improve the signal acquisition in slave channels. Moreover, the Doppler frequency estimated from the master tracking loop could be used to assist the weak signal tracking in the slave loop. The aiding loop structure of the dual antenna receiver is shown in Figure 2.

Since the assistance is from the main loop to the slave loop, the transfer function and the performance of the main loop are the same as those of a standard loop described in the previous section. The slave loop is a multiple inputs single output (MISO) system, due to the Doppler assistance from the master loop. Therefore the performance of the slave loop is different from the traditional one. In this section, the output phase error is analyzed in details with regard to each error source mentioned above. As described in the last section, the dynamic stress and clock noise prevent loop bandwidth reduction. Thus, the dynamic stress and clock phase noise will be discussed first below, followed by the thermal noise effect. In addition, the differential phase error induced by each error source is also analyzed as a factor, due to its importance of the DGNSS kinematic positioning.

### 3.1. Dynamic Stress Induced by Satellite Motion

Dynamic stress appears when dynamic models in tracking loops cannot model the satellite/user motion properly, for instance, when using a low order tracking loop to track signals which experience high order dynamics. For a static receiver, satellite motion causes a maximum Doppler rate of 0.936 Hz/s [15]. This is a typical low dynamic scenario. Under this condition, the Doppler frequency estimated from the master loop reflects the dynamic of the satellite. Thus, in the dual antenna assistance tracking loop, the Doppler aiding could eliminate the dynamic completely in the slave loop. As a result, a 2nd order loop could track the carrier signal of the slave antenna easily with the assistance from a 3rd order master loop. Besides, the differential phase error caused by the dynamic will be zero due to coherence of the Doppler frequencies.

### 3.2. Clock Phase Noise

The clock phase noise in a receiver can be modeled as colored phase noise. The wave generated by the oscillator can be represented as [1]:

(11) $$Asin\left[\left(\omega_{0}t+\phi_{0}\right)+\left(\phi_{l}+\omega_{l}t+2\pi d\frac{t^{2}}{2}\right)+x\left(t\right)\right]$$

Here the amplitude is as assumed to be a constant. In the phase item, $\left(\omega_{0}t+\phi_{0}\right)$ represents the theoretical phase, and $\left(\phi_{l}+\omega_{l}t+2\pi d\frac{t^{2}}{2}\right)$ is the causal phase error, which is mainly induced by the temperature, magnetism, acceleration, and atmospheric pressure, *etc.*, the last item x(t) represents the stochastic error. In the causal phase error, the frequency and frequency change rate have the same effect as the dynamic, which can be eliminated by the master loop assistance in the slave loop. Thus, only the effect of the stochastic phase error x(t) is considered in this paper.

For the clock phase noise $\theta_{c}\left(s\right)$ in an analogous loop system, it can be easily known from Figure 2 that the output phase noise of the master loop includes two items: one is the $\theta_{c}\left(s\right)$ directly from the NCO, the other is the clock phase noise fed back from the loop output. Hence, the equation below can be derived:

(12) $$\theta_{o1}\left(s\right)=\theta_{c}\left(s\right)-\frac{\theta_{o1}\left(s\right)KF_{1}\left(s\right)}{s}$$

Here $K=K_{d1}K_{o}$. A more concise formula could be expressed as follows:

(13) $$\begin{array}{l} \theta_{o1}\left(s\right)=\frac{s}{s+KF_{1}\left(s\right)}\theta_{c}\left(s\right)\\ =H_{e1}\left(s\right)\theta_{c}\left(s\right) \end{array}$$

This equation is the clock phase noise of an independent loop, from which the clock phase noise variance could be accumulated. Assuming the Power Spectral Density (PSD) of the input clock phase noise is $G_{\phi }\left(\omega \right)$, the phase jitter induced by clock phase noise can be derived by accumulation, which will be in accord with Equation (10).

The phase error induced by clock phase noise of the slave loop can be shown as:

(14) $$\theta_{o2}\left(s\right)=\theta_{c}\left(s\right)-\theta_{o2}\left(s\right)\frac{KF_{2}\left(s\right)}{s}-\theta_{o1}\left(s\right)\frac{KF_{1}\left(s\right)}{s}$$

This first term is the clock phase noise. The second term represents the output feedback after passing through the loop filter and the NCO. The last term is the assistance noise from the master loop filter. Submitting the Equation (13) to the Equation (14), the total output phase noise of the slave loop induced by the clock phase noise could be expressed as:

(15) $$\begin{array}{l} \theta_{o2}\left(s\right)=\frac{s}{s+KF_{2}\left(s\right)}\theta_{c}\left(s\right)-\frac{KF_{1}\left(s\right)}{s+KF_{2}\left(s\right)}\cdot \frac{s}{s+KF_{1}\left(s\right)}\theta_{c}\left(s\right)\\ =\frac{s}{s+KF_{2}\left(s\right)}\left[1-\frac{KF_{1}\left(s\right)}{s+KF_{1}\left(s\right)}\right]\theta_{c}\left(s\right)\\ =\frac{s}{s+KF_{2}\left(s\right)}\cdot \frac{s}{s+KF_{1}\left(s\right)}\theta_{c}\left(s\right)\\ =H_{e1}\left(s\right)H_{e2}\left(s\right)\theta_{c}\left(s\right) \end{array}$$

From the equation above, it can be seen that the phase jitter induced by clock phase noise is equivalent to the output of the clock phase noise, which has passed through the master loop error transfer function $H_{e1}\left(s\right)$ and the slave loop error transfer function $H_{e2}\left(s\right)$ in sequence. Since the two functions are both high pass filters, and the cut-off frequency of the master loop $H_{e1}\left(s\right)\;$ is larger than that of the slave loop $\;H_{e2}\left(s\right)$, then $H_{e2}\left(s\right)$ could be neglected and the result would be the same as Equation (13).

From the analysis above, since output phase errors of the master and slave loops are almost equivalent, the difference should be almost zero. It can also be derived from the *S* domain as follows:

(16) $$\begin{array}{l} \theta_{o1}\left(s\right)-\theta_{o2}\left(s\right)=\frac{s}{s+KF_{1}\left(s\right)}\theta_{c}\left(s\right)-\frac{s}{s+KF_{2}\left(s\right)}\cdot \frac{s}{s+KF_{1}\left(s\right)}\theta_{c}\left(s\right)\\ =\left[H_{e1}\left(s\right)-H_{e1}\left(s\right)H_{e2}\left(s\right)\right]\theta_{c}\left(s\right)\\ =H_{e1}\left(s\right)\left[1-H_{e2}\left(s\right)\right]\theta_{c}\left(s\right)\\ =H_{e1}\left(s\right)H_{2}\left(s\right)\theta_{c}\left(s\right) \end{array}$$

From the equation above, it can be seen that the system from the clock noise to the differential phase noise is equivalent to a cascade system using the master loop error transfer function and the slave loop transfer function. When the cut off frequency of the high pass filter is bigger than the low pass filter, the output should be zero.

### 3.3. Thermal Noise

As shown in Figure 2, the master loop is an independent loop, whose output phase error induced by thermal noise is the same as Equation (8). For the slave loop, neglecting the colored noise such as oscillator, and only considering the thermal noise, the system can be seen as a double input one output system. This can be given as follows:

(17) $$\{\begin{array}{c} \theta_{e1}\left(s\right)=\theta_{i1}\left(s\right)\frac{s}{s+KF_{1}\left(s\right)}\\ \theta_{e2}\left(s\right)=\theta_{i2}\left(s\right)-\theta_{o2}\left(s\right)\\ \theta_{o2}\left(s\right)=\theta_{e2}\left(s\right)\frac{KF_{2}\left(s\right)}{s}+\theta_{e1}\left(s\right)\frac{KF_{1}\left(s\right)}{s} \end{array}$$

Solving these equations, the output phase of the slave loop can be derived as follows:

(18) $$\begin{array}{l} \theta_{\text{o}2}\left(s\right)=\theta_{i2}\left(s\right)\frac{KF_{2}\left(s\right)}{s+KF_{2}\left(s\right)}+\theta_{i1}\left(s\right)\frac{KF_{1}\left(s\right)}{s+KF_{1}\left(s\right)}\cdot \frac{s}{s+KF_{2}\left(s\right)}\\ =\theta_{i2}\left(s\right)H_{2}\left(s\right)+\theta_{i1}\left(s\right)H_{1}\left(s\right)H_{e2}\left(s\right)\\ =\theta_{i2}\left(s\right)H_{2}\left(s\right)+\theta_{i1}\left(s\right)H_{1}\left(s\right)\left(1-H_{2}\left(s\right)\right) \end{array}$$

**Figure 3:**
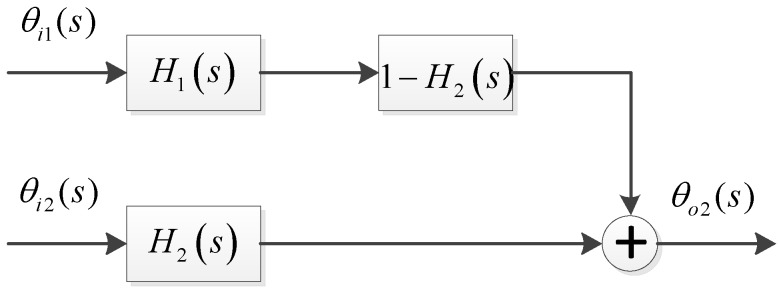
Equivalent structure of the slave tracking loop.

In the equation above, *H*_1_(*s*) and *H*_2_(*s*) are the loop transfer functions of the master and slave loops respectively. *H_e_*_2_(*s*) is the error transfer function of the slave loop. From the equation, an equivalent double input single output system can be obtained:

From Equation (18) and Figure 3, besides the thermal noise from the slave antenna, the noise along with the assistance from the master loop should also be considered. The noise from the Doppler aiding is equivalent to the noise from the master antenna going through a new sub system, whose transfer function is:

(19) $$H_{d}\left(s\right)=H_{1}\left(s\right)\left(1-H_{2}\left(s\right)\right)=\frac{KF_{1}\left(s\right)}{s+KF_{1}\left(s\right)}\cdot \frac{s}{s+KF_{2}\left(s\right)}$$

**Figure 4:**
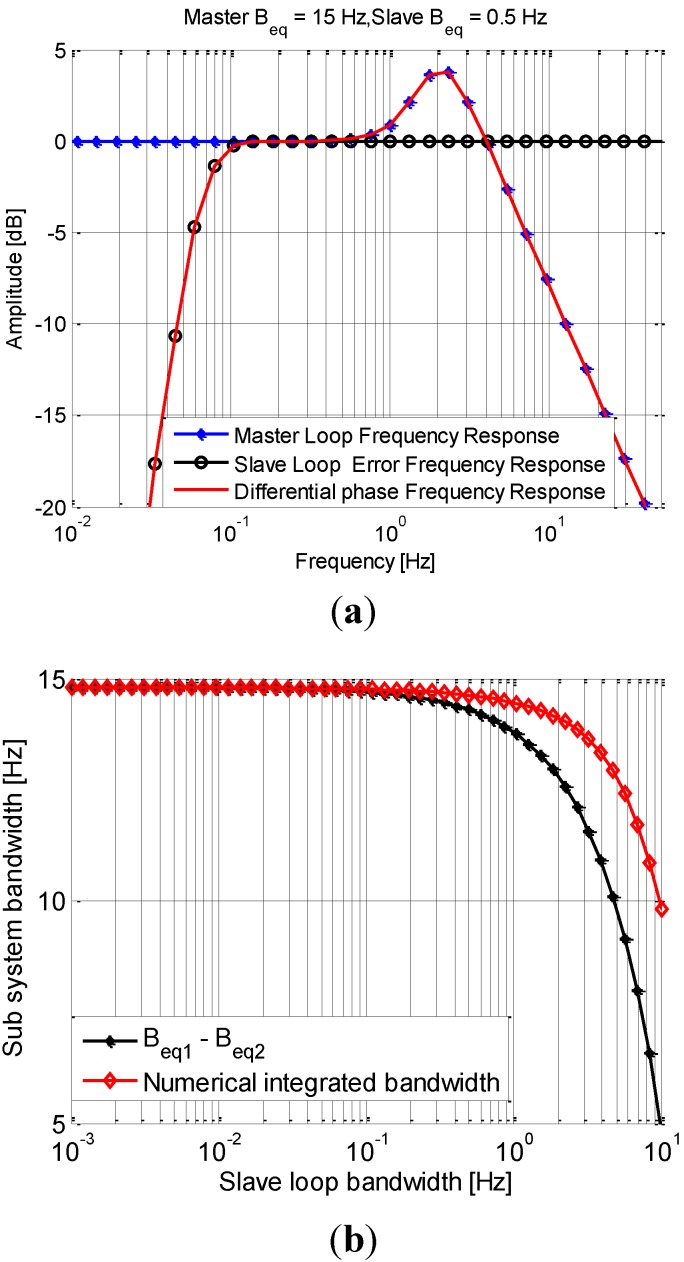
Frequency responses and the bandwidth of the sub system. (**a**) Frequency responses of *H*_1_(*s*), *H*_e2_(*s*) and *H_d_*(*s*); (**b**) Bandwidth of the sub system.

The frequency response of the sub-system are compared with those of the master and slave loops in Figure 4. In this figure, the bandwidth of the master loop *B*L1 is 15 Hz, $\omega_{\text{n}1}=12.5\;rad/s$, and the bandwidth of the slave loop is *B*L2 is 0.5 Hz, $\omega_{\text{n}2}=0.4167\;rad/s$. From Figure 3 and Figure 4a, the new sub system is a cascaded by a low pass filter with a higher cutoff frequency and a high pass filter with a lower cutoff frequency. Thus, it is equivalent to a band pass filter with the cutoff frequencies as the two cutoff frequencies from the two cascaded filters. Its bandwidth approximately is equal to *B*_L1_ − *B*_L2_. Mathematically, the equivalent noise bandwidth of the master loop, slave loop, as well as the sub system can all be computed by Equation (4). A numerical integration method is used to get the actual bandwidth of each filter. Figure 4b shows the bandwidth values of the system with the approximated equation *B*_L1_ − *B*_L2_ and the numerical method. It can be found that the actual bandwidth is very close to the value from the approximation equation *B*_L1_ − *B*_L2_ when the slave loop bandwidth is less than 1 Hz. Thus, the approximate value *B*_L1_ − *B*_L2_ is used to compute the carrier phase jitters below for simplicity.

According to Equation (18), phase jitter induced by the thermal noise at the output of the slave loop comprises two parts as well: the former is equivalent to the noise from the slave antenna at the output of the slave loop, and the latter is equivalent to the noise from the master antenna at the output of the sub-system *H_d_*(*s*), whose bandwidth is *B*_L1_−*B*_L2_. Assuming that both ideal equivalent filter system gains are equivalent to 1, the thermal noise jitter of the slave loop output can be derived from Equations (5), (8) and (18):

(20) $$\begin{array}{l} \sigma_{t2}^{2}=\frac{N_{02}}{2\pi }\int_{0}^{\infty }|H_{1}(\omega ){|}^{2}d\omega +\frac{N_{01}}{2\pi }\int_{0}^{\infty }|H_{d}(\omega ){|}^{2}d\omega \\ =\frac{N_{02}}{2}\cdot 2B_{L2}\cdot {\left|H_{1-ideal}(\omega )\right|}_{G}^{2}+\frac{N_{01}}{2}\cdot 2B_{Ld}\cdot {\left|H_{d-ideal}(\omega )\right|}_{G}^{2}\\ =\frac{B_{L2}}{C/N_{02}}\left(1+\frac{1}{2T_{coh2}C/N_{02}}\right)+\frac{\left|B_{L1}-B_{L2}\right|}{C/N_{01}}(1+\frac{1}{2T_{coh1}C/N_{01}}) \end{array}$$

where *N*_01_/2 and *N*_02_/2 represent the equivalent PSD of the noise received from the master and slave antennas, and the *B_Ld_* is the equivalent sub system bandwidth.

Likewise, the differential phase between outputs of the two loops can be easily derived from Equation (18) and Figure 2 as follows:

(21) $$\begin{array}{l} \theta_{d}\left(s\right)=\theta_{o1}\left(s\right)-\theta_{\text{o}2}\left(s\right)\\ =\theta_{o1}\left(s\right)-\theta_{i2}\left(s\right)H_{2}\left(s\right)-\theta_{o1}\left(s\right)H_{e2}\left(s\right)\\ =\theta_{o1}\left(s\right)\left(1-H_{e2}\left(s\right)\right)-\theta_{i2}\left(s\right)H_{2}\left(s\right)\\ =\theta_{i1}\left(s\right)H_{1}\left(s\right)H_{2}\left(s\right)-\theta_{i2}\left(s\right)H_{2}\left(s\right)\\ =\left[\theta_{i1}\left(s\right)H_{1}\left(s\right)-\theta_{i2}\left(s\right)\right]H_{2}\left(s\right) \end{array}$$

**Figure 5:**
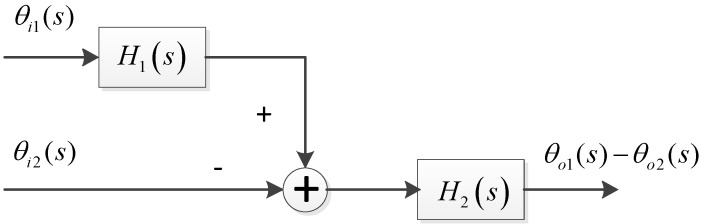
Equivalent structure of the differential phase system.

The equivalent structure of the system is shown in Figure 5:

Because the bandwidth of the slave loop is much smaller than that of the master loop, the effect of thermal noise from the assistance to the differential phase is mainly depends on the slave loop. Mathematically, the differential phase jitter induced by thermal noise can be derived as follows:

(22) $$\begin{array}{l} \sigma_{d}^{2}=\frac{N_{02}}{2\pi }\int_{0}^{\infty }|H_{2}(\omega ){|}^{2}d\omega +\frac{N_{01}}{2\pi }\int_{0}^{\infty }|H_{2}(\omega ){|}^{2}d\omega \\ =\frac{N_{02}}{2}\cdot 2B_{L2}{\left|H_{2-ideal}(\omega )\right|}_{G}^{2}+\frac{N_{01}}{2}\cdot 2B_{L2}\cdot {\left|H_{2-ideal}(\omega )\right|}_{G}^{2}\\ =\frac{B_{L2}}{CN_{02}}\left(1+\frac{1}{2T_{coh2}CN_{02}}\right)+\frac{B_{L1}}{2B_{L1}CN_{01}}\left(1+\frac{1}{2T_{coh1}CN_{01}}\right)\cdot 2B_{L2}\\ =\frac{B_{L2}}{CN_{02}}\left(1+\frac{1}{2T_{coh2}CN_{02}}\right)+\frac{B_{L2}}{CN_{01}}\left(1+\frac{1}{2T_{coh1}CN_{01}}\right) \end{array}$$

It can be seen from the equation above, the assisted loop structure outperforms the independent loop in the accuracy of the differential phase.

## 4. Simulation Results

Semi-analytical simulation techniques [16] were adopted to show the performance of the proposed dual antenna loop, compared to the two independent loops. Since the phase errors in a single loop have been researched in many references, the performance simulation in this paper mainly includes the phase jitters in the slave loop and the differential phase as the results of the dynamic, thermal noise, and clock phase noise.

### 4.1. Dynamic Stress

**Figure 6:**
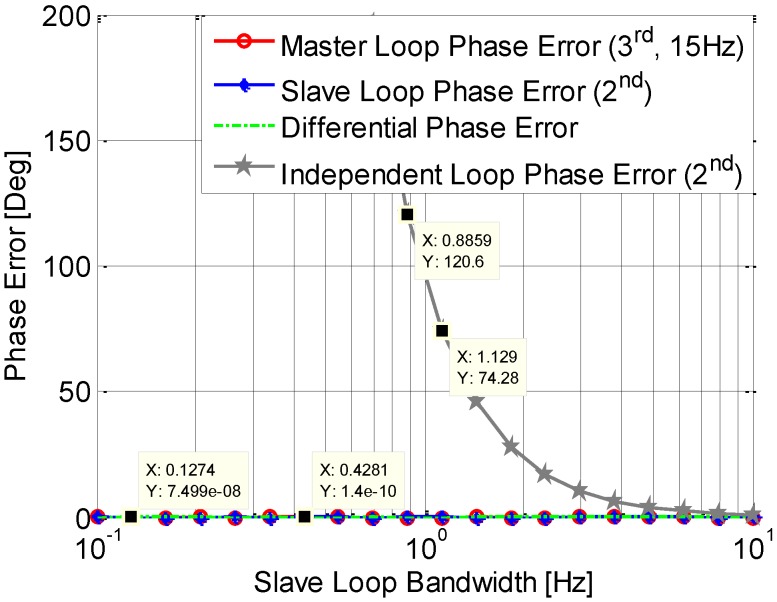
Phase jitter induced by dynamic.

The phase error induced by the dynamics is examined by simulation. In the simulation, the signal dynamic is set to be 0.936 Hz/s; the master loop is a 3rd order loop, with a bandwidth of 15 Hz, and the slave loop used is a 2nd order loop with a variable bandwidth from 0.1 Hz to 10 Hz. It is shown in Figure 6 that the steady state phase errors of the master loop and slave loop, as well as the single differential phase error between them, are all almost zeros. Due to the Doppler aid from the 3rd order loop used in the master loop, the slave tracking loop can track the signals with low bandwidth. In contrast, the phase error of an independent 2nd order loop increases when the bandwidth reduces. Especially, when the bandwidth is below 2 Hz, the steady state phase error is more than 45°, which could result in a loss of lock. This phenomenon is in accord with the analysis from Equation (9).

### 4.2. Clock Phase Noise

**Figure 7:**
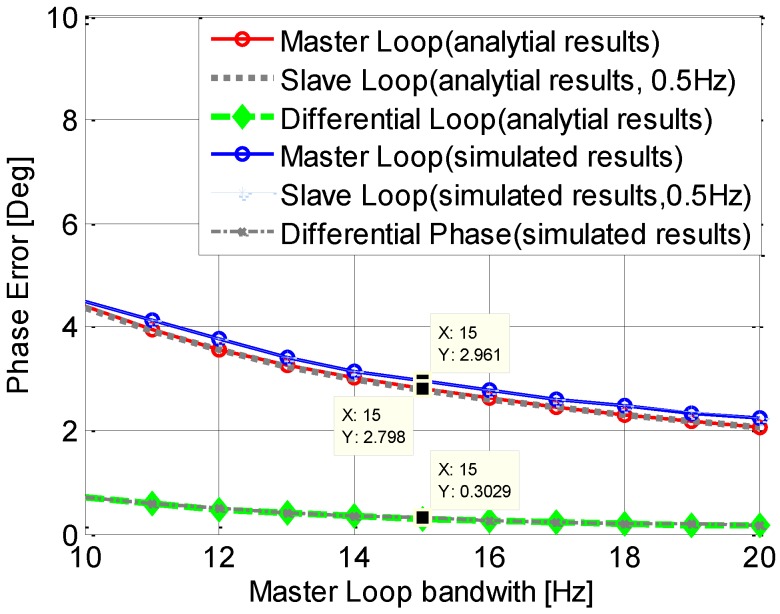
Comparisons of the analytical and simulated results.

Two main types of clock phase noises are discussed in this section, namely Allan Deviation phase noise and the vibration induced phase noise [3]. In the simulation, the parameters for the TCXO are chosen as: h_0_ = 1e-21, h__1_ = 1e-20, h__2_ = 2e-20, and the parameters of the OCXO is set to be: h_0_ = 2.51e-26, h__1_ = 2.51e-23, h__2_ = 2.51e-22. In order to validate the equations provided in Section 3, the phase jitters induced by Allan Deviation phase noise (TCXO) both in the analysis using Equations (15) and (16) and a simulation are given in Figure 7. In this simulation, the master loop bandwidth is changed from 10 Hz to 20 Hz, and the slave loop bandwidth is 0.5 Hz. As depicted in the figure, phase error of the master loop is almost equivalent to that of slave loop, and they increases as the master loop bandwidth decreases. The differential loop phase error is very small when the master loop bandwidth is large enough, such as 15 Hz. More importantly, the analytical results are similar to the simulated results, which could illustrate the Equations (15) and (16) are right.

**Figure 8:**
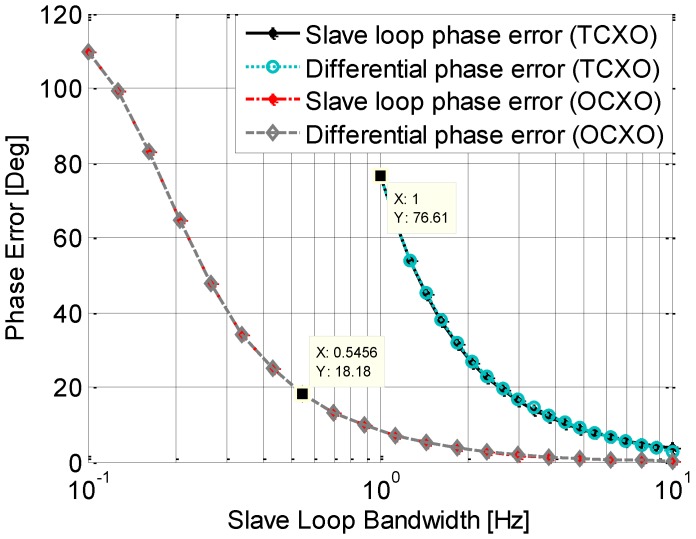
Phase jitter induced by Allan Deviation phase noise with an independent loop.

**Figure 9:**
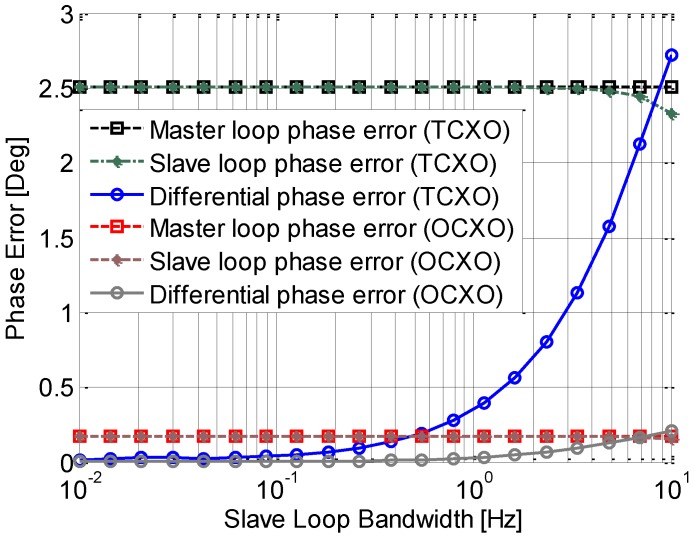
Phase jitter induced by Allan Deviation phase noise with an assisted loop.

Figure 8 and Figure 9 show the phase jitters induced by Allan Deviation phase noise with the independent and assisting tracking loop structure respectively. The master loop is a 3rd order loop with a fixed bandwidth of 15 Hz, and the slave loop is a 2nd order loop with a variable a bandwidth ranged from 0.1 Hz to 10 Hz. The phase jitter of the independent tracking loop is shown in Figure 8. Due to the fixed bandwidth, the phase jitter of the master loop is a constant, which is shown in the Figure 9. In accord with the analysis in the previous section, the phase error of an independent loop becomes larger as the bandwidth decreases. As expected, the OCXO outperforms TCXO, especially for a narrow bandwidth, such as 1 Hz. Another phenomenon shown in this figure is that when the bandwidth is small than a threshold (2 Hz for TCXO and 1 Hz for OCXO), the phase jitter increases rapidly, which could result in a loss of loop. It also can be seen from the figure that the differential phase jitter is almost the same as that of the slave loop. That is because the phase jitter caused by the master loop is much smaller due to its relative large bandwidth.

Figure 9 shows the results of the assisting tracking loop. It is shown that the phase jitter of the two loops is almost equivalent. This matches the analysis presented above. Different from the independent loop structure, the differential phase jitter becomes smaller with the decrease of the bandwidth. It is almost zero when the slave loop bandwidth is smaller than 1 Hz. This means the slave loop could track the signal stably even with a small bandwidth, which is less than 1 Hz. This is because the results are closer to the theoretical results when the cut-off frequencies of the two loops become further apart. It can be also seen that in this assisting tracking loop structure, the differential phase jitter with the two types of oscillators are both very small, especially when the slave loop bandwidth is smaller than 1 Hz. That means the contributions of the two oscillators are almost identical in the differential phase error, the requirement of the oscillator in the receiver is greatly reduced.

**Figure 10:**
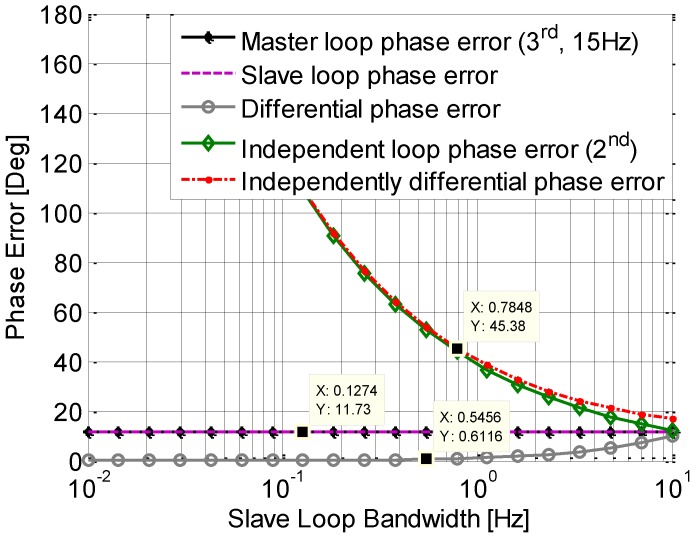
Phase jitter induced by oscillator vibration.

Based on the same method, vibration induced phase jitters of the independent and assisting tracking loop are analyzed and simulated in this paper. The oscillator’s sensitivity is set to be typical value *K*_g_ = 1e-9 1/g, and a constant PSD of vibrations of *G*_g_ = 0.05 g^2^/Hz is used with the frequency under 20 Hz ignored. Similar with Allan Deviation induced phase jitter, it can be seen from Figure 10 that vibration induced phase jitter becomes larger with decrease of the bandwidth. It can exceed the tracking threshold (45°) [2] when the bandwidth is smaller than 1 Hz. The differential phase jitter mainly depends on the slave loop due to its much smaller bandwidth. However, in the assisting tracking loop structure, phase jitters of the two loops are almost equivalent, and the differential phase jitter is almost zero. Hence, the vibration induced phase jitter in the slave loop could be eliminated by the assistance from the master loop. Therefore the slave loop in this structure could adopt a very small bandwidth to suppress thermal noise. Meanwhile, the differential phase jitter could be greatly reduced, resulting in a more precise relative positioning.

### 4.3. Thermal Noise

**Figure 11:**
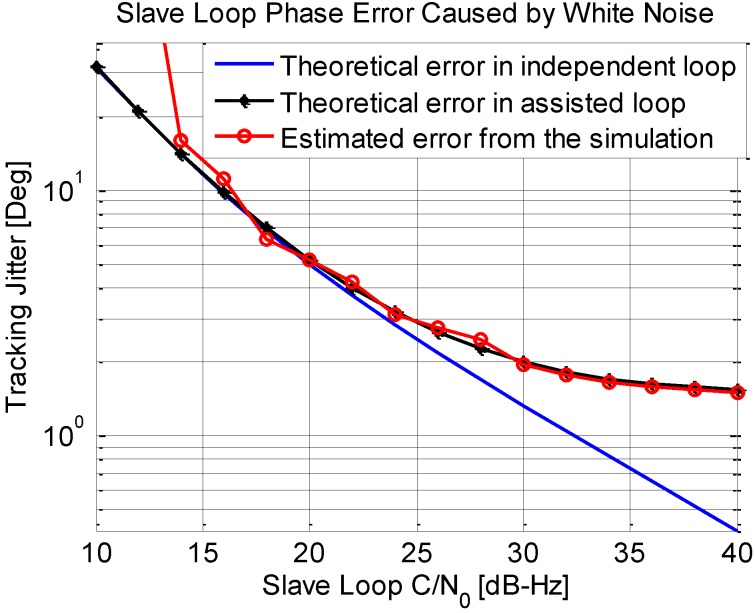
Phase jitter of slave loop output induced by thermal noise.

Figure 11 shows the phase jitters of the slave loop induced by thermal noise. In the simulation, the carrier noise ratio of the signal received from the master antenna is set to be 45 dB-Hz, while the signal received from the slave antenna varies from 10 dB-Hz to 40 dB-Hz. The master loop is a 3rd order loop with a bandwidth of 15 Hz, while the slave loop is a 2nd order loop with bandwidth of 0.5 Hz. The coherent integration time in both loops is 10 ms. As shown in Figure 10, the phase jitter of the slave loop with the new structure is larger than that in the independent loop, since extra noise originates from the assistance from the master loop. This phenomenon becomes more apparent when the C/N_0_ values of the two loops are close, and become less apparent when the difference of the C/N_0_ values is larger. This can be explained by Equation (20). Given that the C/N_0_ of the master antenna signal is firmed and high enough, when the C/N_0_ of the salve antenna is low, the error mainly depends on the slave loop. However, when the C/N_0_ of the salve antenna signal is high enough, the total error variance is about two times of the error of an independent loop.

**Figure 12:**
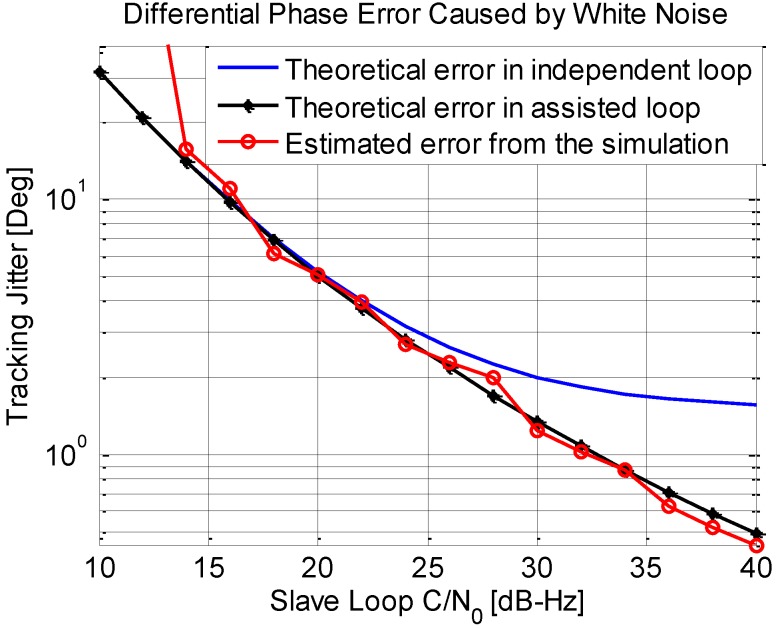
Phase jitter of differential phase output induced by thermal noise.

The differential phase jitters of the two structures are shown in Figure 12. It can be clearly seen that the differential phase jitter with assisting tracking loop is smaller than the one in the independent loop, especially when carrier noise ratio values of both loops are similar. That is because of the second item of Equation (22) dominants under this condition, compared to that in the independent loop structure. While, the differential phase jitter mainly depends on the slave loop error with the *C/N*_0_ of the slave decline, resulting in a similar performance between the two structures. Due to analysis above, however, it should be highlighted that a very small bandwidth such as 0.5 Hz cannot be used in the independent loop due to the satellite motion caused by dynamic and TCXO instability as described above.

## 5. Test Results with a Software Receiver

The proposed dual antenna tracking loop structure was realized in a software GPS receiver. The performance of the assisted loop was evaluated and compared with an independent one. The dual antenna IF signals were collected from a hardware simulator by a two channel collector with a complex sampling frequency 5.0625 MHz and zero central frequency. The baseline between the two antennas is set to be 265.8718 m. Hereinto, the carrier noise ratio of the master antenna signal is set to be 48 dB-Hz, while that of the slave antenna signal varies along with the time from 48 dB-Hz to 18 dB-Hz. In the assisted loop structure, the master antenna signal was tracked by a 3rd order loop with a bandwidth of 15 Hz. A 2nd order loop with a bandwidth of 0.5 Hz was used to track slave antenna signal. The coherent integration time in the master and slave loops are separately 1 ms and 20 ms.

**Figure 13:**
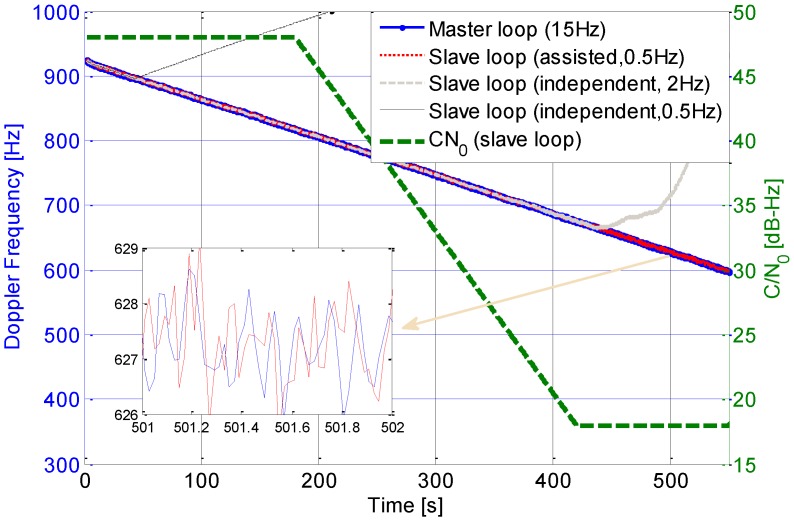
Tracking performance comparisons in a software GPS receiver.

Performance of the assisted carrier tracking loop is shown in Figure 13. In the experiment, two 3rd order loops with bandwidths of 2 Hz and 0.5 Hz were used, respectively, to track the signals from the slave antenna independently. As shown in the figure, the independent loop with a bandwidth of 2 Hz could track the slave loop with some signal attenuation, which, however, couldn’t be maintained in the weak signal environment. When the bandwidth of 0.5 Hz was used, in order to enable successful frequency pull-in of the slave PLL, a bandwidth of 2 Hz was used for the first 50 s. After the pull-in stage, the bandwidth was changed to 0.5 Hz. As depicted in the figure, the loop with the bandwidth of 0.5 Hz lost lock when the signal was strong enough. This indicates that the bandwidth was too narrow for carrier tracking likely due to clock phase noise in this case. In contrast, the proposed slave tracking loop can track signal when the *C/N*_0_ values decreases to 18 dB-Hz. The enlarged details show that the Doppler frequency of the slave loop is similar to that of the master loop, even at the *C/N*_0_ 18 dB-Hz, which means that the dynamic stress and the clock phase noise of the slave antenna signal are eliminated by the assistance from the master loop.

## 6. Discussion and Conclusions

In order to track the GNSS signal in a degraded signal environment, an assisted tracking structure loop was used in a dual antenna receiver. The analytical formulations of the noise jitters of the proposed tracking loop were provided. The performance of the proposed tracking loop was analyzed in terms of dynamic stress, thermal noise jitter, and clock phase noise jitter. Simulation results show that the dynamic and clock noise in the slave loop could be eliminated with the assistance from the master loop. This ensures that a very narrow bandwidth could be used to suppress noise and improve the tracking performance of weak signals. Test results with a software receiver and signal collected from the hardware signal simulator show that the proposed tracking loop allow weak carrier signal tracking (18 dB-Hz) with a narrow bandwidth of 0.5 Hz for static applications. The integration of an IMU and the proposed dual antenna to provide Doppler aid for user motion compensation will be considered in future work.
